# Genital self-mutilation in psychosis: a two-case report and literature review on psychopathological mechanisms and management

**DOI:** 10.1192/j.eurpsy.2026.11324

**Published:** 2026-07-31

**Authors:** F. Zaouali, I. Anes

**Affiliations:** 1 Emergency department and mobile emergency and resuscitation service, Tahar Sfar University Hospital, Mahdia; 2 Outpatient psychiatric consultation, Hadj Ali Soua Regional Hospital, Ksar Hellal, Monastir, Tunisia

## Abstract

**Introduction:**

Genital self-mutilation (GSM) refers to socially unacceptable, self-inflicted injuries to the external genitalia, usually without suicidal intent. Though rare, GSM is a dramatic clinical presentation, often associated with severe psychiatric disorders, particularly psychosis.

**Objectives:**

This study aims to explore the psychopathological underpinnings and multidisciplinary management of GSM through two clinical cases and a systematic review of the literature.

**Methods:**

We report two cases of GSM in patients diagnosed with schizophrenia spectrum disorders, and conducted a narrative literature review. We searched PubMed and PsycINFO using the keywords: “genital self-mutilation”, “psychosis”, “schizophrenia”, and “self-injury”. Inclusion criteria were case reports, case series, and reviews published in English or French over the last 30 years.

**Results:**

The first case involved a 25-year-old man with chronic schizophrenia, who attempted penile amputation. He believed his erections were externally induced and represented a threat. He was hospitalized under involuntary status, received emergency urological care, and was stabilized with antipsychotic treatment. The second case concerned a 54-year-old woman with paranoid schizophrenia. She inflicted injuries to her labia majora, claiming she was possessed by a demon who assaulted her at night, a situation she found religiously intolerable. She underwent local wound care and psychopharmacological adjustment. A total of 58 articles were identified, of which 26 met the inclusion criteria and were analyzed in terms of clinical characteristics, psychiatric diagnoses, and therapeutic approaches. In the literature, GSM was predominantly observed in males (over 80%), with schizophrenia being the most frequent diagnosis. Common psychotic themes included religious delusions, sexual guilt, and command hallucinations. Management always required a combined urological and psychiatric approach. Surgical outcomes were variable, but psychiatric stabilization reduced recurrence risk.

**Conclusions:**

GSM is a rare but severe manifestation of psychosis, often rooted in delusional thinking related to sexuality and religious beliefs. Early psychiatric diagnosis and a collaborative approach with urology are critical for patient recovery. A better understanding of its symbolic meaning in psychosis may help clinicians prevent such extreme behaviors.

**Disclosure of Interest:**

None Declared

